# Examining the continuity of modifiable cancer-risk behaviors from youth into adulthood through prospective longitudinal studies: A scoping review

**DOI:** 10.1016/j.socscimed.2025.118534

**Published:** 2025-09-02

**Authors:** Mackaully L. Parada, Jeremy Horn, Christopher Cambron

**Affiliations:** The University of Utah, College of Social Work, 395 1500 E, Salt Lake City, UT, 84112, United States of America

**Keywords:** Cancer prevention, Alcohol, Tobacco, Diet, Physical activity, Longitudinal

## Abstract

**Objective:**

Modifiable health behaviors including tobacco and alcohol use, poor diet, and low physical activity increase risk for developing multiple cancers. Longitudinal research suggests that risky behaviors initiated in youth may persist into adulthood. This scoping review maps prospective longitudinal studies examining the continuity of these behaviors from youth into adulthood.

**Methods:**

Four electronic databases were searched for prospective longitudinal studies on the continuity of tobacco use, alcohol consumption, poor diet, and low physical activity from youth (<18) into adulthood (≥18). The scoping review was guided by the Preferred Reporting Items for Systematic reviews and Meta-Analyses extension for Scoping Reviews (PRISMA-ScR) and methodology outlined by the Joanna Briggs Institute. Three independent reviewers used Covidence review management software for screening and data extraction.

**Results:**

Seventy-one studies met inclusion criteria. The majority of studies examined alcohol use (58 %) and tobacco use (24 %), with fewer studies addressing low physical activity (6 %) or poor diet (4 %). Over 90 % of studies reported continuity of behaviors from youth into adulthood. Persistence was most consistently observed for alcohol and tobacco use, with limited evidence supporting continuity of poor diet and low physical activity.

**Conclusions:**

This review summarized available prospective longitudinal research on the continuity of health risk behaviors from youth into adulthood. While there is evidence for the continuity of youth alcohol and tobacco use into adulthood, notable research gaps exist for poor diet and physical activity, limiting our understanding of how these behaviors track across development. Implications for future cancer research are discussed.

## 1. Introduction

Cancer is a leading cause of death worldwide, responsible for one in six deaths globally (World Health Organization [WHO, 2024). The International Agency for Research on Cancer (IARC) estimates that there were approximately 20 million new cancer cases and nearly 10 million deaths reported in 2022, with projections of 35 million new cases by 2050 (Bray et al., 2024). As of January 2022, approximately 18 million individuals were living with cancer in the United States. The National Cancer Institute (NCI) estimates that over two million additional individuals will receive a new cancer diagnosis by the end of 2024 (NCI, 2024). Recent reports suggest that diagnoses of early onset cancer before age 50 steadily rose from 1990 to 2019 and are projected to increase by up to 30 % by 2039 largely due to the compounding influence of unhealthy lifestyles (Zhao et al., 2023). The WHO estimates that one-third of cancer-related deaths are directly attributable to modifiable health behaviors including tobacco and alcohol use, poor diet, and low physical activity (WHO, 2025).

According to the American Cancer Society (ACS), tobacco use is responsible for approximately 30 % of all cancer deaths and is causally implicated in at least 16 cancers (ACS, 2022; Cancer Research UK, 2023). Additionally, alcohol consumption, poor diet, excess body weight, and low physical activity account for 18 % of cancer diagnoses and 16 % of cancer-related deaths (ACS, 2022). The increased cancer risk associated with poor diet and low physical activity is in large part thought to be attributable to excess body fat or obesity. Among young adults, poor diet and obesity have been linked to earlier development of malignancies (Berger, 2019). Importantly, these modifiable cancer risk behaviors often occur in tandem within individuals leading to a substantial increase in overall risk (Champion et al., 2018; Noble et al., 2015; Silva et al., 2019; Vermeulen-Smit et al., 2015). As such, cancer prevention approaches would benefit from simultaneously promoting multiple healthy behaviors rather than addressing specific risk behaviors independently.

The ACS and other public health advocacy groups regularly publish guidelines for individual and community action to promote healthy behaviors and reduce unhealthy behaviors linked to cancer risk (Rock et al., 2020). Health promotion and behavior change interventions have demonstrated the ability to support smoking cessation (Golechha, 2016; Patnode et al., 2021), reduced alcohol intake (Fergie et al., 2018; O’Connor et al., 2018), improved diet (Bull et al., 2018; Chen et al., 2020) and increased physical activity (Gilchrist et al., 2024; Schwartz et al., 2019), though effectiveness may differ across countries, populations, and socioeconomic groups (Lustria et al., 2013; WHO, 2024). Research exploring the efficacy of these interventions presents a range of individual, community, and society-level approaches with strong evidence of directly reducing or delaying cancer incidence. Interventions include client-centered counseling (i.e. Motivational Interviewing), peer education, urban planning strategies, dissemination of information via influential community channels (healthcare providers, religious organizations, etc.), media advocacy, and public health/legislative approaches, among others. These preventive interventions, however, have historically focused on adults (Rock et al., 2020).

There is increasing recognition that cancer prevention among youth represents an essential part of the cancer care continuum (Brindis, 2017). This recognition results in part from the growing empirical literature indicating that early exposure to tobacco smoke is associated with increased risk for multiple later life cancers independent of other known risk factors (Clarke and Joshu, 2017). Recent evidence also suggests that both poor diet and childhood obesity may be associated with increased risk for diagnosis of multiple cancers and overall cancer mortality (Hidayat et al., 2018; Lei et al., 2020; Nuotio et al., 2022). Other studies have shown mixed associations between and youth obesity and later life risk for breast cancer (Hidayat et al., 2017; Okasha et al., 2003) or prostate cancer (Robinson et al., 2008). A recent review indicated inconsistent evidence for an association between youth physical activity and later life cancers (Clarke and Joshu, 2017) despite some evidence that increased physical activity across a lifetime may reduce risk for breast, colon, and endometrial cancer (Hidayat et al., 2019). Few empirical studies have examined associations between youth alcohol use and later life cancer. Despite the well-established association between adult alcohol use and cancer risk, empirical studies involving youth alcohol use are yet to provide clear evidence of a link.

The extent to which youth health behaviors are associated with later life cancers is largely predicated on the continuity of those behaviors into adulthood. While there are well-established theoretical perspectives suggesting that health behaviors in youth may persist into adulthood, patterns also may shift over time in response to changing life circumstances and developmental transitions. Many youth experiment with tobacco and alcohol use without becoming consistent, heavy users as adults (Brown et al., 2008; Mathers et al., 2006). Additionally, dietary and physical activity patterns may change as youth move from high school into adulthood and take on new professional and familial roles (Winpenny et al., 2020). That said, individuals tend to remain embedded in systems and relationships known to shape influence health behaviors (Champagne, 2010; Lodi-Smith and Roberts, 2007; Umberson et al., 2010). For example, family and peer relationships often directly influence individual-level health behaviors, while broader socioeconomic, community, and cultural factors contextualize individual-level behaviors by shaping opportunities and norms related to health behavior in the short- and long-term (Bronfenbrenner, 1979; Office of Disease Prevention and Health Promotion [ODPHP], 2020; World Health Organization [WHO], 2024, 2025). As such, continuity of health behaviors from youth into adulthood is expected. A deeper understanding of the extent to which youth health behaviors continue into adulthood can provide important information for cancer prevention efforts at both the individual and community levels.

Life Course Theory explains the development of health behaviors over time as shaped by developmental transitions, personal decisions, historical and geographic contexts, social roles, and interpersonal relationships (Elder, 1998). This perspective would suggest that behaviors initiated in youth may persist into adulthood because they become embedded within influential social and institutional systems including family, school, employment, and community structures. The concept of “health behavior careers” serves as a complimentary sociological perspective that frames behaviors like substance use and inactivity as patterned trajectories that are shaped by social structures and individual meaning-making, which often unfold through identifiable stages (initiation, maintenance, desistance, relapse, etc.; Becker, 1963; Cockerham, 2005). In addition to individual autonomy, other social and cultural factors contribute to how these careers develop over time (Blumer, 1969; Umberson et al., 2010). Such frameworks may advance and contextualize the understanding of behavioral continuity by recognizing that health behaviors are dynamic and evolve alongside key domains and transitions of the life course.

Understanding initiation, maintenance, withdrawal, and long-term persistence as interconnected phases of substance use trajectories can offer a more comprehensive lens for interpreting continuity. For example, neurobiological and psychosocial mechanisms that contribute to the initiation and maintenance of risky behaviors in youth may also play a role in their persistence into adulthood and the challenges associated with cessation. Though this review focused on the continuity of modifiable health behaviors, situating behavioral persistence within the broader framework of addiction science may enhance understanding of why some youth transition out of risky behaviors while others do not, further informing prevention efforts.

The goal of this study is to summarize the current evidence base on the continuity of youth tobacco and alcohol use, diet, and physical activity to inform cancer prevention efforts. A preliminary search was conducted across three different databases – JBI Database of Systematic Reviews and Implementation Reports, Cochrane Library, and PROS-PERO – on February 16, 2023 to determine the existence and extent of scoping or systematic reviews related to the topic of continuity of modifiable cancer risk behaviors. To date, no such review has been performed to map the existing literature on the youth-to-adulthood trajectory of tobacco use, alcohol consumption, poor diet, and low physical activity across the transition from youth to adulthood. Scoping reviews use a systematic and iterative methodology to identify and synthesize a body of literature (Mak and Thomas, 2022), which can be valuable for researchers, physicians, and educators to rapidly identify the extent of the research that exists around modifiable youth health patterns that have been established as precursors to cancer later in life. Additionally, this methodology facilitates an outline of strengths, weaknesses, and knowledge gaps which is useful for developing recommendations for future research, practice, and policy.

The current scoping review was designed to describe the extent and nature of prospective longitudinal studies that address the continuity of four cancer risk behaviors from youth into adulthood: tobacco use, alcohol use, poor diet, and low physical activity. For the purposes of this research, “continuity” referred to the behavior being recorded in childhood or adolescence (prior to 18 years of age) and then after the participant is an adult (18 years of age or older). Engagement in the behaviors was determined by either participant self-report, interview responses, survey responses, questionnaire responses, or biomarker data gathered by the source researchers.

## 2. Materials and methods

This scoping review was guided by the reporting recommendations proposed by Shamseer et al. (2015) in the Preferred Reporting Items for Systematic Review and Meta-Analysis Protocols (PRISMA-P). The review was grounded in the Arskey and O’Malley (2005) framework and applied methodology outlined by the Joanna Briggs Institute (Peters et al., 2020). PRISMA-S (Preferred Reporting Items for Systematic Reviews and Meta-Analysis extension for Searches) is a 16-item checklist that guided the reporting of the search process (Rethlefsen et al., 2021). The review protocol was registered on the Open Science Framework (OSF) prior to data extraction (https://doi.org/10.17605/OSF.IO/X8V64). The results of the review are presented using the PRISMA-ScR (Preferred Reporting Items for Systematic Reviews and Meta-Analyses extension for Scoping Reviews), a 20-item checklist designed to serve as a guideline for reporting on the core concepts and key items unearthed in the review (Tricco et al., 2018).

### 2.1. Eligibility criteria

A PCC (Population/Concept/Context) framework informed the development of the main constructs and ultimately guided the research question’s eligibility criteria, search strategy, and data charting tool (Peters et al., 2020). The review included prospective longitudinal studies that examined the continuity of health-risk behaviors from childhood (ages 0–17 at baseline) across the transition to adulthood (age 18+). Eligible studies were not required to implement a specific statistical method but had to clearly report outcomes that demonstrated whether behaviors persisted across this developmental transition. Studies included both descriptive and inferential analyses, however those that used latent class modeling, growth trajectory analyses, or other advanced longitudinal modeling techniques were excluded because they generally assess patterns of change across multiple timepoints rather than explicitly measuring the presence of the same behavior at two distinct life stages (once during youth (<18) and again during adulthood (≥18)).

Studies must have reported on at least one of the following health-risk behaviors: tobacco use, alcohol consumption, poor diet, and/or low physical activity. Inclusion was not limited to a time-range for publication and both national and international settings were considered. Studies that were not in English with no translation available were excluded due to funding limitations. Grey literature search was not conducted. Systematic reviews, meta-analyses, and scoping reviews discussing related topics were reviewed, however they were not considered eligible for the purposes of this study.

Of note, several well-known longitudinal studies of adolescent health — including National Longitudinal Study of Adolescent to Adult Health (Add Health) and Project EAT (Eating and Activity over Time) — were identified during the screening process. However, these studies were not included in this review because their baseline samples included participants who were already 18 years of age or older at the first wave of data collection, which did not meet our inclusion criteria of youth (<18 years) at baseline. Additionally, though the review was not limited to U. S. datasets and considered eligible studies globally, a large portion of the literature originates from Western nations. As such, findings may be more reflective of U.S. public health systems and social contexts.

### 2.2. Information sources and search strategy

The research team conducted a search of electronic databases including MEDLINE via PubMed, EMBASE (Elsevier), CINAHL (Complete Ebscohost), and APA PsycINFO (Ebscohost). The search was executed in three stages as recommended by the Joanna Briggs Institute. First, an initial search was conducted using the main criteria that were iteratively developed throughout the pilot searches of two online databases: MEDLINE via PubMed and CINAHL. One search strategy example can be found in Appendix A. The second stage of the search involved identifying keywords from the titles and abstracts of studies that were selected in the preliminary search. The keywords and index terms were adapted and applied to all four databases included in the study. Each individual database included appropriate MeSH (Medical Subject Headings) or equivalent terms, Boolean operations, and proximity operators for construction of key terms. As part of the search strategy, reviewers also manually reviewed the reference lists of all full-text articles selected for inclusion to identify any additional studies that met the eligibility criteria.

### 2.3. Study records: data management, selection process, and data collection process

**Fig. 1.**
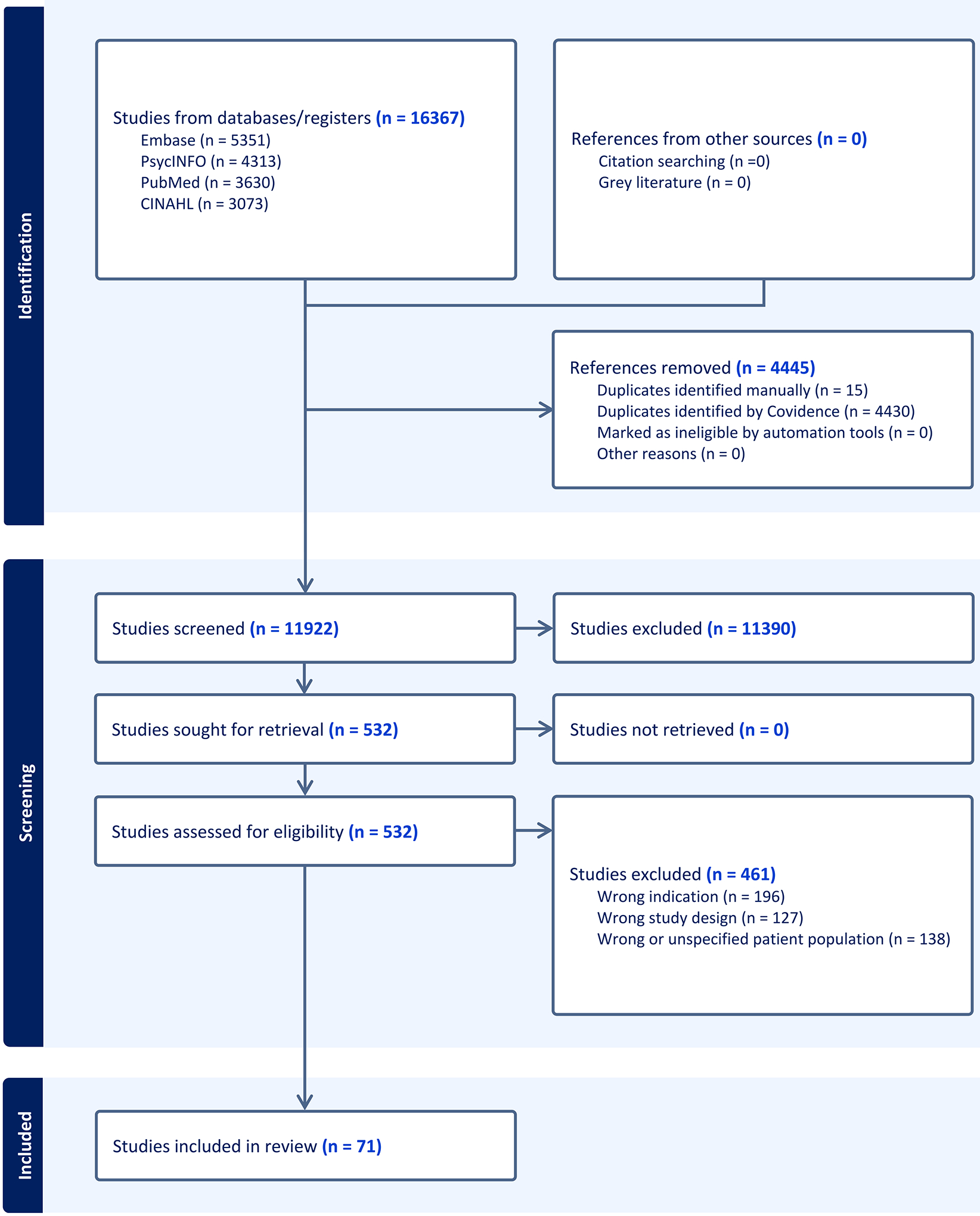
PRISMA flow chart.

The initial search results were saved in Endnote X20 (Clarivate) and then exported into Covidence (Veritas Health Innovation) for data management, including removal of duplicates, screening, and extraction. Two independent reviewers screened titles and abstracts and selected prospective longitudinal studies that were consistent with our population, concept, and context (PCC) format. Studies selected during the initial screen were then evaluated by two independent reviewers in the full-text screening phase, and studies that did not meet the inclusion criteria were excluded. Disagreements that arose regarding the inclusion of a study were resolved by a third reviewer, or with discussion and consensus between the reviewers. A PRISMA-ScR flow diagram is provided outlining our process, documenting the number of articles reviewed/included/excluded at each stage, and providing explanations for excluded studies (Fig. 1). Data were charted in table format using guidance from the Joanna Briggs Institute data extraction instrument (Peters et al., 2020). The piloted data charting tool was developed using Microsoft Excel and structured around our PCC format. Charting includes key information such as author(s), year of publication, country, population, context, and outcomes/findings.

### 2.4. Outcomes and prioritization

Using the charting tool designed to identify, characterize, and summarize the extracted data, the research team descriptively mapped the results from the included sources (Peters et al., 2020). Levac et al.’s (2010) three-step framework guided the data analysis/reporting procedure: (1) analyze the data, (2) report the results, and (3) apply meaning to the results. Reporting consisted of study characteristics, outcomes, and themes, as well as the strengths and knowledge-gaps in the existing literature. This led into the study’s third aim, which was to examine the implications of the review within the contexts of continued research, policy changes, and improved clinical practice.

## 3. Results

The search of MEDLINE via Pubmed, EMBASE, CINAHL, and PsycINFO yielded a total of 16,367 hits. From these records, 4445 duplicates were removed either manually or via Covidence deduplication software, resulting in 11,922 articles. Two independent reviewers conducted a comprehensive title/abstract screen of the included studies, and conflicts that arose due to either (1) disagreements around inclusion vs. exclusion or (2) selection of different exclusion justification, were resolved by a third reviewer. A total of 532 studies were moved to the full-text phase, 461 of which were excluded by two independent reviewers for one of the following reasons: other indication, other or unspecified patient population, or other study design. Conflicts were resolved via consensus or by a third reviewer and data were extracted from the 71 studies that remained for analysis. The flow chart depicted in Fig. 1 illustrates the screening and inclusion/exclusion processes.

### 3.1. Article characteristics

The 71 studies examined in this review were conducted across 12 countries: Australia (n = 8; 11 %), Canada (n = 2; 3 %), Denmark (n = 2; 3 %), Finland (n = 3; 4 %), Germany (n = 1; 1 %), New Zealand (n = 2; 3 %), Norway (n = 6; 8 %), Portugal (n = 1, 1 %), Sweden (n = 6; 8 %), the United Kingdom (n = 5; 7 %), and the United States (28; 38 %). Four studies were conducted across two countries: Australia and New Zealand (n = 1, 1 %), Australia and Norway (n = 1, 1 %), and Australia and the United States (n = 2; 3 %). Dates of publication ranged from 1986 to 2023 with data collection ranging from 1968 to 2021. Participant ages at baseline ranged from 4 months through 17 years old; the oldest participants with follow-up ages reported were 43 years old (some studies used “adults” or “year *x* of the study” to indicate follow-up age categories). The majority of the studies collected data at more than 2 timepoints (n = 58; 82 %). Two studies had less than 100 participants and the remaining studies ranged from 139 to 10,833 participants. Eleven studies (16 %) utilized nationally representative samples. All studies considered at least one of the following modifiable cancer risk behaviors: tobacco use (n = 17; 24 %), alcohol use (n = 41; 58 %), poor diet (n = 3; 4 %), and low physical activity (n = 4; 6 %). Five studies (7 %) examined both alcohol and tobacco use, and 1 study (1 %) addressed all four behaviors.

### 3.2. Outcomes

Table 1 displays a summary of findings from the data extraction. Each study was assessed as to whether there was continuity of the modifiable behavior(s) from youth (<18 years old at baseline) into adulthood (≥18 years old at follow-up). Sixteen of the 17 studies (94 %) reported that tobacco use in youth was associated with continued tobacco use in adulthood, with only one study reporting mixed results. Among these studies, rates of continuity were consistently high. For example, Chassin et al. (1996) reported that approximately 59 % of individuals who smoked during adolescence were still smoking in adulthood. More recently, Dutra and Glantz (2018) found that even occasional smoking in adolescence (1–5 cigarettes per month) tripled the odds of smoking in adulthood.

Regarding alcohol use, 44 out of 47 studies (94 %) reported continuity from youth into adulthood. Two studies reported mixed results, and one study did not observe a continuity of alcohol use from youth into adulthood. Continuity rates for alcohol use followed similar trends to that of tobacco use across adolescence into adulthood. For example, Bonomo et al. (2004) reported that youth who began regular drinking in adolescence had 2–3 times the odds of developing alcohol dependence in adulthood, and Degenhardt et al. (2013) reported that 61 % of males and 42 % of females who engaged in heavy binge drinking in adolescence continued this behavior into adulthood.

Three out of the four studies examining poor diet reported continuity from youth into adulthood and all five studies that measured low physical activity reported continuity from youth into adulthood, however continuity estimates were notably lower. For example, Anderssen et al. (2005) found that only 25 % of adolescents maintained consistent physical activity from youth into adulthood; 20 % of youth reported an increase in activity from baseline (age 13) to Year 8 (age 21), while 53–58 % reported a decrease.

## 4. Discussion

This scoping review summarized current evidence from prospective longitudinal studies on tobacco and alcohol use, poor diet, and low physical activity and specifically assessed the extent to which studies have reported a continuity of these four cancer risk behaviors from youth to adulthood. Overall, support for continuity of tobacco and alcohol use from youth to adulthood was identified. Support for continuity of poor diet and low physical activity was noted but few studies sought to examine continuity of these behaviors. Results reveal potential gaps in the literature that could advance understanding of behavioral continuity and inform prevention efforts. First, there is a need for more globally representative longitudinal data to explore behavioral patterns across different cultural and socioeconomic contexts. Second, including mediators and moderators would facilitate a better understanding of the mechanisms behind behavioral persistence vs. desistence and clarify the processes that drive health patterns. Finally, more research exploring the co-development of multiple health behaviors could advance our knowledge of behavior clustering and better inform integrated prevention strategies. Each cancer risk behavior is discussed below with additional recommendations for future research.

### 4.1. Tobacco use

The continuity of tobacco use from youth to adulthood was most commonly represented by statistical models reporting that smoking cigarettes as a youth predicted smoking cigarettes regularly or having nicotine dependence in adulthood. The large quantity of studies addressing this question reflects the relative ease to which cigarette smoking can be identified along with the broad range of harms directly attributable to cigarette smoking (ACS, 2022). While cigarette smoking among adults and initiation of cigarette use among youth has declined substantially in recent decades, specific demographic groups including lower socioeconomic status, LGBTQ+, and some racial/ethnic minority populations have continued to smoke at higher rates requiring more focused interventions to mitigate cancer risks (https://pubmed.ncbi.nlm.nih.gov/29384589/). Additionally, the more recent widespread adoption of electronic cigarettes (e-cigarettes) or vape devices among youth presents a new delivery method for potential carcinogens. While e-cigarettes are largely considered safer than traditional combustible cigarettes and may support cigarette smoking cessation among adults, recent studies suggest that e-cigarettes may also present direct carcinogenic risks for heavy users and increase the likelihood of future cigarette smoking among youth (Baenziger et al., 2021; Glantz and Bareham, 2018; Sahu et al., 2023). Future prospective longitudinal studies with both e-cigarette and dual e-cigarette/combustible cigarette users examining later life incidence of lung and other cancers is warranted.

### 4.2. Alcohol use

The continuity of alcohol use from youth to adulthood was most commonly represented by The continuity of alcohol use from youth to adulthood was most commonly represented by statistical models reporting any alcohol use as a youth predicting later life binge drinking, Alcohol Use Disorder, heavy drinking, or other constructs denoting problematic or consistent drinking behavior. While youth consistently report less alcohol use in recent years, approximately 30 % of US high school students report any past 30-day alcohol use (CDC, 2022a,b) and greater than 60 % of adults report consuming alcohol (Schaeffer and DeSilver, 2024). Broad indicators like “any past year use” or “total lifetime use” are commonly reported in longitudinal studies, though it is important to consider that accurately measuring alcohol consumption remains challenging due to factors like recall bias, social desirability, and the variability of alcoholic beverages and serving sizes. Despite the widespread use of alcohol, the consistency of these results in this review on the continuity of alcohol use from youth into adulthood, and the established research on alcohol as a carcinogen, there has been limited examination of the potential contribution of youth alcohol use to later life cancer risk. Future studies on this topic are needed.

### 4.3. Continuity of poor diet

Few studies included in this review investigated the continuity of poor diet from youth into adulthood. Part of this lack of research is likely due to unclear definitions of which specific habits, patterns, and trends indicate a poor diet. For example, one study examined soft drink consumption and low fruit and vegetable consumption, which was identified as “unhealthy behavior” (Thuen et al., 2015). Another study measured sugar-sweetened beverage consumption as a key factor in a “poorer diet” (Bolt-Evensen et al., 2018). A third study analyzed free sugar intake and dietary glycemic load due to their evidence-based association with metabolic health issues (Marinho et al., 2020), and an additional study measured fast food intake as a key contributor to low “dietary quality” (Lipsky et al., 2015). Second, the lack of longitudinal studies examining poor dietary habits could be related to continually evolving nutritional standards. For example, since 1980, the U.S. Department of Agriculture has released new standards for American food consumption every 5 years (U.S. Department of Agriculture [USDA] & U.S. Department of Health and Human Services [DHHS], 2020). As such, it may be difficult for researchers to consistently define a “poor diet” over the length of time necessary to conduct longitudinal research. The primary objectives of the Dietary Guidelines for Americans 2020–2025 provide some guidance on this issue with the goal of lowering the risk for chronic diseases (USDA & DHHS, 2020). The USDA has developed a 100-point scale designed for adherence tracking, providing an objective measurement tool that may make a construct of overall poor diet more straightforward to measure. Future investigations on adherence to dietary guidelines across transition from youth to adulthood and the contribution of non-adherence to later life cancer risk are needed.

### 4.4. Continuity of low physical activity

Few prospective longitudinal studies on the continuity of low physical activity from youth to adulthood were identified by this review. This is partly because most studies analyzed isolated physical activity behaviors that weren’t necessarily indicative of a low level of physical activity overall. For example, screen time is a commonly analyzed behavior, and while there may be some associations between screen time and low physical activity, screen time is not inherently a measure of low physical activity. Other studies examined participation in certain physical activities, physical performance metrics, or anthropomorphic metrics. These common measures do not give a clear indication of whether a person’s lifestyle was generally low in physical activity and therefore, did not fit our study parameters. All included studies displayed a continuity of low physical activity from youth into adulthood. These studies described low physical activity as “sedentary” participants (Anderssen et al., 2005), those who did “not meet the criteria” for being physically active (Li et al., 2016), “inactives” (Parker et al., 2022), “low” physical activity participants (Suppli et al., 2013), and “lack of physical activity” (Thuen et al., 2015) respectively. These studies were unique in that they described a specific threshold for low overall physical activity that could be identified as a health risk behavior. There is a need for future prospective longitudinal research on groups who specifically engage in a low overall level of physical activity. Future studies should examine overall level of physical activity across the transition from youth to adulthood to help illuminate potential associations with later life cancer risk.

### 4.5. Strengths and limitations

This is the first scoping review examining prospective longitudinal studies of the continuity of four cancer risk behaviors from youth to adulthood. This study utilized PRISMA guidelines for scoping reviews to ensure fidelity in study methods and enhanced replicability. Utilizing Covidence to organize and structure our review, three independent reviewers were able to work asynchronously thus reducing opportunity for bias to influence inclusion of studies. The inclusion of international studies enhances the significance of the findings, providing broader implications on a global scale. Consistent findings across multiple countries suggest that geographic or national context may have a limited influence on the continuity of these cancer risk behaviors from youth into adulthood, at least within the regions represented in the included studies.

There are several limitations to consider. First, restrictions around search criteria or database indexing may have excluded high quality studies and limited the generalizability of the findings. Excluding grey literature and non-English/untranslated papers may omit relevant studies and international research, creating bias in the findings toward published works and English-speaking countries or contexts. Differences in how authors define age groups serves as another limitation. For example, the Add Health and Project EAT studies define adolescence as a period from 12 to 18 years old, whereas this review defines “youth” on a pre-18-year-old timeline. According to the definition outlined in the review, studies were not included if they had participants who were already 18 at baseline; while justified according to clear inclusion criteria, this may have inevitably eliminated other eligible longitudinal data.

Additionally, this review’s focus on continuity between discrete youth and adult timepoints led to a decision to exclude studies that used statistical methods like trajectory or latent class analysis. As these methods help track patterns of change over time, this exclusion may limit our understanding of more nuanced behavioral trajectories. Further, this scoping review did not conduct a formal appraisal of quality on articles that met search criteria. Therefore, it is important to acknowledge that the included studies may be subject to selection or information biases, especially considering the reliance on self-reported data and diverse sampling strategies. A future systematic review is needed to differentiate the high and low-quality prospective longitudinal studies on cancer-risk behaviors and a future meta-analysis is needed to define effect sizes for continuity of risk behaviors. Finally, it is important to consider potential publication bias that may result from researchers not publishing null findings on the continuity of cancer risk behaviors from youth into adulthood.

Although this review highlights continuity in individual modifiable cancer risk behaviors, the clustering of multiple behaviors from youth into adulthood was not commonly examined in the included studies. Existing literature shows that multiple risk behaviors often co-occur across adolescence and adulthood (Champion et al., 2018; Noble et al., 2015; Silva et al., 2019; Vermeulen-Smit et al., 2015). As such, future research should investigate how these behaviors jointly persist across developmental stages, thus improving targeted prevention efforts and supporting more holistic cancer risk reduction strategies.

The geographic distribution of included studies represents another important limitation. The vast majority of studies were conducted in Western countries, particularly the United States, Australia, and European nations. This limits the generalizability of findings, as the continuity of health behaviors may differ in non-Western contexts. Future longitudinal research is needed in underrepresented regions like Asia, Africa, and Latin America to better understand behavior patterns across diverse populations and inform culturally relevant cancer prevention strategies.

## 5. Conclusion

This scoping review maps the current literature on the continuity of common, modifiable youth cancer risk behaviors into adulthood. Results can inform both life-course cancer risk research and youth prevention programming. Researchers should consider the continuity of multiple cancer risk behaviors simultaneously across the transition from youth to adulthood to better understand the clustering of risk. Life-course epidemiology frameworks can help guide the assessment of both critical periods for risk exposure and accumulation of risks across the transition into adulthood (Ben-Shlomo and Kuh, 2002). Further, considering these trajectories in the context of “behavioral careers” offers a useful lens for interpreting how health-related actions are shaped by broader structural and cultural forces (Becker, 1963; Blumer, 1969; Cockerham, 2005).

Pediatricians, oncologists, prevention practitioners, and public health officials should emphasize the importance of collectively preventing youth tobacco use, delaying or preventing youth alcohol use, and improving diet and physical activity among youth as explicit cancer prevention strategies. Given that most cancer do not emerge until later in life, clear messaging on the accumulation of risk across the life course is needed. Although our findings primarily reflect Western contexts, global health organizations have emphasized that cultural, economic, and political differences may shape youth health behaviors in distinct ways, calling for future research among underrepresented regions to inform culturally responsive cancer prevention strategies. The overall goal of this review is to equip researchers, educators, providers, and policymakers with the knowledge needed to guide future research and youth-based prevention programs to reduce lifetime cancer risk. To support future research and policy efforts, Table 2 outlines key recommendations based on the findings of this review. These recommendations address identified research gaps and suggest directions for advancing longitudinal research on health behavior continuity across the life course.

## Data Availability

No data was used for the research described in the article.

## Appendix A. Search Strategy

Search Strategy: MEDLINE via PubMed.

**Table:**

Population	1	(“Child” [Mesh] OR “Adolescent” [Mesh] OR “youth*”[Title/Abstract] OR “child*”[Title/Abstract] OR “adolescen*”[Title/Abstract] OR “teen*”[Title/Abstract])
	2	(“adult*”[Title/Abstract])
Concept: Modifiable cancer-risk behavior	3	(“Tobacco use”[Mesh] OR “Tobacco use”[Title/Abstract:~2] OR “cigar*”[Title/Abstract] OR “smok*”[Title/Abstract])
	4	(“Alcohol drinking”[MeSH Terms] OR “alcohol drinking”[Title/Abstract:~2] OR “alcohol use”[Title/abstract:~2])
	5	(“Diet, Food, and Nutrition”[MeSH Terms] OR “diet”[Title/Abstract] OR “food”[Title/abstract] OR “nutrition”[Title/Abstract])
	6	(“Exercise”[MeSH Terms] OR “Physical Activities”[MeSH Terms] OR “physical activity”[Title/Abstract:~2] OR “physical activity”[Title/abstract:~2] OR “Sedentary Behavior” [MeSH Terms] OR “sedentary behavior”[Title/Abstract:~2] OR “inactive*”[Title/Abstract])
Context	7	(“Prospective Studies”[Mesh]) OR (“prospective study”[Title/Abstract:~2] OR “prospective studies”[Title/Abstract:~2] OR “prospective cohort”[Title/Abstract:~2] OR “prospective cohorts”[Title/Abstract:~2] OR “prospective methods”[Title/Abstract:~2])
	Final Search: (1 AND 2) AND (3 OR 4 OR 5 OR 6) AND (7)	

## Appendix B. Summary Tables

**Table 1:** Summary of findings.

Author and Year	Country	Behavior(s) investigated	Age(s) at baseline Sample	Age(s) at follow-up Sample	Total number of participants Sample	Continuity of risk behavior
Andersen et al. (2003)	DNK	Alcohol	14–15	19	729	Continuity observed
Anderson et al. (2013)	USA	Alcohol	15	25	447	Continuity observed
Anderssen et al. (2005)	NOR	Low PA	13.3	21	557	Continuity observed
Andersson and Magnusson (1988)	SWE	Alcohol	14–17	18–24	703	Continuity observed
Andersson et al. (1989)	SWE	Alcohol	15–17	18–23	570	Continuity observed
Bagnall et al., 1991	UK	Alcohol	15–16	24–25	778	Continuity not observed
Bailey et al. (2021)	AUS & USA	Alcohol	15	25	1707	Continuity observed
Berg et al. (2019)	SWE	Alcohol	16	43	1010	Continuity observed
Boden et al. (2020)	NZL	Alcohol	14–16	35	962	Continuity observed
Boisvert et al. (2017)	CAN	Alcohol	16	22	289	Continuity observed
Bolt-Evensen et al. (2018)	NOR	Poor diet	11.8	26.5	437	Continuity not observed
Bonomo et al. (2004)	AUS	Alcohol	year 9	20.7	1601	Continuity observed
Bonomo (2005)	FIN	Alcohol	14	42	369	Continuity observed
Brière et al. (2013)	USA	Alcohol	16.6	30	816	Mixed results
Brook et al. (2013)	USA	Tobacco	14.1	32.3	815	Continuity observed
Buu et al. (2015)	USA	Alcohol; Tobacco	9th grade	24	850	Continuity observed; Continuity observed
Cable and Sacker (2008)	UK	Alcohol	16	30	10833	Continuity observed
Chassin et al. (1996)	USA	Tobacco	17	29	4035	Continuity observed
Degenhardt et al. (2013)	AUS	Alcohol	14.9	29	821	Continuity observed
Duncan et al. (1997)	USA	Alcohol	13.03	19–26	480	Continuity observed
Dutra and Glantz (2018)	USA	Tobacco	12 to 16	26–30	5240	Continuity observed
Enstad and Kjeldsen, 2018	NOR & AUS	Alcohol	14.6	18.9	458	Continuity observed
Georgiades and Boyle (2007)	CAN	Tobacco	12 to 16	“Young Adult”	1282	Continuity observed
Green et al. (2011)	USA	Alcohol	16	42	702	Continuity observed
Grevenstein et al. (2015)	DEU	Alcohol; Tobacco	14	24	318	Continuity observed; Continuity observed
Grøtvedt et al. (2013)	NOR	Tobacco	15.9	18.7	1395	Continuity observed
Guo et al. (2000)	USA	Alcohol	10	21	808	Continuity observed
Guttmannova et al. (2011)	USA	Alcohol	10	33	692	Continuity observed
Guttmannova et al. (2012)	USA	Alcohol	10	33	706	Continuity observed
Huurre et al. (2010)	FIN	Alcohol	15.9	32	2091	Continuity observed
Irons et al. (2015)	USA	Alcohol	14.8	25.29	1328	Continuity observed
Janson (1999)	SWE	Tobacco	12	36	146	Continuity observed
Jefferis et al. (2005)	UK	Alcohol	16	42	8520	Continuity observed
Jester et al. (2019)	USA	Tobacco	6 to 8	18–20	78	Continuity observed
King and Chassin (2007)	USA	Alcohol	13	25	395	Continuity observed
Kisely et al. (2020)	AUS	Tobacco	14	21	3758	Continuity observed
Krange and Pedersen (2001)	NOR	Tobacco	13.7	20.8	488	Mixed results
Li et al. (2016)	USA	Low PA	16.2	“1st year after high school”	2785	Continuity observed
Li et al. (2017)	USA	Alcohol	15	28	3444	Continuity observed
Lipsky et al. (2015)	USA	Poor diet	10th & 11th grade	“adults”	2785	Continuity observed
Magee and Connell (2021)	USA	Alcohol	17	30	498	Continuity observed
Marinho et al. (2020)	PRT	Poor diet	13	21	1764	Continuity observed
Mason and Spoth (2011)	USA	Alcohol	11.34	21	161	Continuity observed
Meza et al. (2023)	USA	Alcohol	14	21	988	Continuity observed
Moore et al. (2009)	AUS	Alcohol	14.9	24.1	1520	Mixed results
Newcomb et al. (1986)	USA	Alcohol	7th-9th grades	21.79	722	Continuity observed
Newcomb and Bentler (1986)	USA	Alcohol; Tobacco	7th-9th grades	“year 9” of the study	654	Continuity observed
Norström and Pape (2012)	NOR	Alcohol	15.5	28.6	1751	Continuity observed
Novak et al. (2007)	SWE	Tobacco	16	30	1044	Continuity observed
Parker et al. (2022)	AUS	Low PA	16.87	“2-year follow-up”	852	Continuity observed
Patton et al. (2006)	AUS	Tobacco	14.9	24.1	1943	Continuity observed
Pitkänen et al. (2008)	FIN	Alcohol	14	42	347	Continuity observed
Rogers et al. (2021)	USA	Alcohol; Tobacco	14.49	26.14	1399	Continuity observed; Continuity observed
Selya et al. (2016)	USA	Tobacco	15.7	24	746	Continuity observed
Silins et al. (2018)	AUS & NZL	Alcohol	13	30	807 to 9453	Continuity observed
Simons-Morton et al. (2016)	USA	Alcohol	10th grade	“First year after high school”	2659	Continuity observed
Stanton et al. (1996)	AUS	Tobacco	9	18	937	Continuity observed
Stanton et al. (2005)	NZL	Tobacco	11	21	1037	Continuity observed
Stephenson et al. (2023)	UK	Alcohol	13.5	24	5160	Continuity observed
Strong et al. (2016)	USA	Tobacco	15–16	42–23	703	Continuity observed
Stueve and O’Donnell, 2007	USA	Tobacco	8th grade	19.7	538	Continuity observed
Suppli et al. (2013)	DEN	Low PA	15	27	561	Continuity observed
Sylvestre et al. (2020)	CAN	Alcohol	12.7–17.1	20–24	787	Continuity observed
Thuen et al. (2015)	NOR	Alcohol; Tobacco; Low PA; Poor diet	13	30	983	Continuity observed; Continuity observed; Continuity observed; Continuity observed
Toumbourou et al. (2014)	USA & AUS	Alcohol	15	21.1	2309	Continuity observed
Van De Ven et al. (2010)	AUS	Tobacco	9th school year	24–25	1520	Continuity observed
Viner and Taylor (2007)	UK	Alcohol	16	30	4911	Continuity observed
Virtanen et al. (2015)	SWE	Alcohol	16	42	30	Continuity observed
Waller et al. (2019)	USA	Alcohol	11	22	139	Continuity observed
Windle and Windle (2012)	USA	Alcohol; Tobacco	15.54	23.5	827	Continuity observed; Continuity observed
Zaso et al. (2020)	USA	Alcohol	16	20	1778	Continuity observed

**Table 2:** Summary of recommendations for future research and practice.

Recommendation Topic	Explanation
Global representation	Longitudinal studies should be conducted in underrepresented regions to inform culturally responsive cancer prevention policies
Diet and physical activity	More research is needed regarding the longitudinal continuity of nutrition and physical activity, as they are far less studied compared to other modifiable health risk behaviors that contribute to cancer development (such as tobacco and alcohol use)
Behavior clustering	There is a need for further examination around the co-development of multiple risk behaviors to support more holistic cancer risk reduction strategies
Continuity/change mechanisms	Including mediators and moderators in longitudinal models would facilitate a better understanding of the mechanisms behind behavioral persistence vs. desistence and clarify the processes that drive health patterns
Sociological and life course framing	The Life Course Theory and sociological concepts like “behavior careers” should be applied more explicitly to longitudinal research around modifiable cancer risk behaviors to support a contextual understanding of behavior patterns
Policy and prevention	Findings related to behavioral continuity should inform education and public health guidelines for early intervention and prevention
